# The use of Tissue PatchTM to Reduce the Duration of Air Leak Following Lung Volume Reduction Surgery.

**DOI:** 10.1186/1749-8090-10-S1-A13

**Published:** 2015-12-16

**Authors:** Alexandra Monaghan, Vijay Joshi, Sridhar Rathinam

**Affiliations:** 1 University of Leicester, Leicester, Leicestershire, LE1 7RH, UK; 2 Department of Thoracic Surgery, Glenfield Hospital, University Hospitals of Leicester, Leicester, Leicestershire, UK

## Background/Introduction

Prolonged post-operative air leak is a recognised complication in patients receiving lung volume reduction surgery (LVRS). Some patients are transferred to a portable flutter-valve bag to facilitate discharge. TissuePatchTM is a synthetic absorbable self-adhesive film which acts as an adjunct to minimise air leak.

## Aims/Objectives

Our aim was to see whether the use of TissuePatchTM would reduce post-operative air leak and the subsequent need for a drain in LVRS patients.

## Method

We retrospectively analysed LVRS cases over a two year period performed by a single surgeon to minimise procedural heterogeneity. Patients were divided into two groups; group 1 received Tissue PatchTM as the staple line adjunct and group 2 did not.

## Results

There were 26 cases in total (one excluded due to in hospital death); group 1=12 (2= bilateral procedures, 10=upper lobe procedures, median age 65), group 2=13 (all unilateral, all upper lobe procedures, median age 63). The median length of stay was 15 for both groups (p = 0.40). The median duration of air leak was 13 days for group 1 and 18 days for group 2 (p = 0.95). Only 2/12 (16%) in group 1 did not have full resolution of air leak and drain removal prior to discharge and were placed on a portable flutter-valve bag compared to 5/13 (38%) in group 2 (p = 0.64).

## Discussion/Conclusion

We have observed a reduced trend in the number of patients being discharged with persistent air leak following LVRS with the concomitant use of Tissue PatchTM. A larger study is indicated which may demonstrate significant results.

